# Reliability of Joint Position Sense and Force Sense Measurements in Children with Developmental Coordination Disorder

**DOI:** 10.3390/jfmk11010035

**Published:** 2026-01-15

**Authors:** Anna Gogola, Piotr Woźniak, Zenta Piscova, Anna Rubika, Liene Lukjaņenko, Irēna Kaminska, Rafał Gnat

**Affiliations:** 1 Institute of Physiotherapy and Health Sciences, Academy of Physical Education, 40-065 Katowice, Poland; aniagogola@op.pl; 2 Developmental Neuro-Motor Stimulation Institute International, 43-600 Jaworzno, Poland; 3 Doctoral School of Physical Culture Sciences, University of Physical Culture, 31-571 Krakow, Poland; piotr.wozniak@dream-motion.pl; 4 Faculty of Natural Sciences and Healthcare, Department of Healthcare, Daugavpils University, LV-5401 Daugavpils, Latvia; zentapiscova@inbox.lv (Z.P.); anna.rubika@du.lv (A.R.); liene.lukjanenko@du.lv (L.L.); irena.kaminska@du.lv (I.K.)

**Keywords:** developmental coordination disorder, joint position sense, force sense, proprioception, reliability

## Abstract

**Background:** Quantitative assessment of proprioception in children with Developmental Coordination Disorder (DCD) is limited by methodological variability and the lack of developmentally appropriate protocols. Joint position sense (JPS) and force sense (FS) assessments are commonly used in adults; however, their reliability in pediatric populations has not been sufficiently established. The objective of this study was to evaluate the intra- and inter-rater reliability of adapted JPS and FS protocols in children with DCD and to determine whether the observed reliability supports the use of these methods in experimental research. **Methods:** A repeated-measurements reliability research design was employed. Twenty-eight children aged 10–15 years (mean age 12.86 years), with a mean body mass of 43.68 kg and a mean height of 149.32 cm, and with medically confirmed DCD, completed four proprioceptive tests: joint angle reproduction and differentiation, and force reproduction and differentiation. Absolute errors were calculated for each trial. Reliability was assessed using intraclass correlation coefficients (ICC2*,k*), standard error of measurement, and smallest detectable difference. Bland–Altman plots were used to evaluate agreement. **Results:** Reliability across all tests and movement directions ranged from good to excellent. Most ICC values exceeded 0.90, with only a small number falling between 0.86 and 0.90. Although differentiation tasks produced larger absolute errors than reproduction tasks, their reliability remained excellent. Bland–Altman analyses demonstrated acceptable bias, reasonable clustering around the mean difference, and only occasional outliers beyond the limits of agreement. **Conclusions:** The adapted JPS and FS protocols demonstrated high intra- and inter-rater reliability in children with DCD, supporting their use in experimental research.

## 1. Introduction

Over recent decades, physiotherapy and related disciplines, including biomechanics and neurophysiology, have advanced substantially in the quantitative assessment of human motor performance. In adult musculoskeletal research, three-dimensional motion capture is routinely used to quantify spinal mobility, pelvic orientation, and complex motor behaviors [1,2,3]. Ultrasonography is widely applied to assess skeletal muscle architecture and activation, including deep and superficial trunk musculature [4,5,6,7]. Myotonometry [8] and elastography [9] enable quantification of biomechanical tissue properties and their modulation by therapeutic intervention [10]. Electromyography [11,12], together with functional magnetic resonance imaging [13,14], provides complementary information on neural and muscular contributions to movement. A similar trend appears in neurodevelopmental physiotherapy, where assessment priorities are shaped strongly by developmental considerations. Motion capture has been widely employed to quantify mobility and alignment [15], while ultrasonography provides detailed information on muscle morphology and activation [16]. Surface electromyography allows characterization of postural muscular activity [17,18]. The use of inertial measurement units further enhances objective evaluation of sensorimotor control, balance, and fine motor coordination [15].

The evaluation of sensorimotor control using modern technologies is particularly important in pediatric populations, as many neurodevelopmental and musculoskeletal disorders involve impaired sensory integration. Even subtle deficits may present as delayed motor milestones, inefficient postural adjustments, or reduced participation in daily activities [19,20,21]. Developmental Coordination Disorder (DCD), the central focus of this study, represents a condition at the interface of typical and atypical motor development and remains frequently under-recognized. It is estimated to affect up to approximately 6% of school-aged children, with a higher prevalence in boys [21,22]. The mechanisms underlying DCD are multifactorial and may involve molecular and morphological factors. Genetic and epigenetic influences have been implicated, including candidate genes affecting neural development and motor function and DNA methylation differences [23]. Morphological studies report atypical development in motor-related brain regions, including the cerebellum, basal ganglia, and cortical motor areas [24]. Together, these factors may contribute to characteristic motor impairments, altered anticipatory and reactive postural control, atypical muscle activation, and proprioceptive deficits—including joint position sense and force discrimination—leading to impaired balance and reduced motor performance [20,25,26,27,28,29]. These findings underscore the need for developmentally appropriate, precise, and robust assessment protocols for proprioception-based sensory functions in pediatric populations [30].

Investigation of proprioception-based sensory functions, such as joint position sense (JPS) and force sense (FS), in children and adolescents presents distinct developmental and methodological challenges. In early childhood, reliable measurement is limited by reduced comprehension and compliance, necessitating procedures with minimal cognitive demands [31,32,33,34]. Although older children can tolerate adapted adult protocols, many of these protocols remain insufficiently validated and lack reliability data for pediatric populations [30,35]. In addition, proprioceptive functions undergo substantial developmental changes related to neural maturation and shifts in body representation, which must be carefully considered when designing and interpreting test protocols [31,36,37].

Although studies in adults have demonstrated high reliability for JPS and FS measures [38,39], evidence in children remains limited and methodologically variable [20,31]. The lack of standardized, developmentally appropriate assessment protocols restricts diagnostic precision and comparability across studies, highlighting the need for reliable of proprioception-based functions in pediatric populations. This study contributes to the existing literature by providing the first systematic evaluation of the intra- and inter-rater reliability of JPS and FS assessment methods in children with DCD. A further objective is to determine whether the observed reliability supports the use of these methods in evaluating targeted therapeutic interventions aimed at improving proprioceptive function and motor control.

## 2. Materials and Methods

### 2.1. Design and Participants

**Figure 1:**
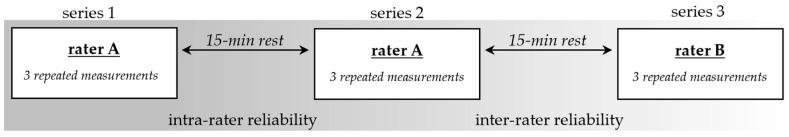
Simplified structure of the joint position sense and force sense measurements, and calculation of the reliability indices.

This study evaluated the reliability of two proprioceptive functions—JPS and FS—using repeated measurements by two raters on the same day (Figure 1). Two specific tests were administered for each function. For JPS, these were the joint angle reproduction test (JPSrep) and the joint angle differentiation test (JPSdif). For FS, the tests included the force reproduction test (FSrep) and the force differentiation test (FSdif). The differentiation tasks represent a novel component in proprioceptive assessment, as they place greater demands on central processing rather than relying solely on peripheral proprioceptive input [32,36,40]. The study protocol was approved by the Committee on Ethics of Scientific Research for Physiotherapists of the Polish Physiotherapy Association (No. 4/06/2025, 11 June 2025).

The minimum required sample size was calculated using Sample Size Calculator 2.0 [41] based on formulas proposed by [42]. The calculation assumed α = 0.05, β = 0.80, a minimum acceptable reliability of 0.70, and an expected reliability of 0.90, resulting in a required sample size of 17 participants. Recruitment was planned to continue for up to one month or until this minimum sample size was reached.

Participants were recruited from local physiotherapy centers specializing in pediatric conditions. Eligibility was verified during an initial meeting and clinical examination conducted by a qualified physiotherapist. Inclusion criteria were: (1) age between 10 and 15 years, (2) a confirmed diagnosis of DCD by a pediatrician, with otherwise typical motor development, (3) full intellectual capacity, and (4) the ability to follow verbal instructions.

Evidence indicates that, although symptoms of DCD may be observable in early childhood, formal diagnosis is most often made during the early school years (typically between 5 and 10 years), while diagnosis before age 5 is uncommon or less reliable [21,43,44]. Therefore, the first inclusion criterion required a DCD diagnosis established between 6 and 10 years of age, corresponding to the early school period. The lower age limit of 10 years was chosen because younger children often have difficulty understanding and following the verbal instructions required for more complex motor tasks, such as the JPS and FS tests used in this study. The upper age limit of 15 years was applied because DCD symptoms are typically most pronounced during childhood and early adolescence; in older adolescents, motor coordination difficulties may become less evident due to developmental maturation and compensatory mechanisms [40,45]. Limiting the sample to this age range ensured that participants were at a developmental stage where DCD-related impairments are most observable and where task demands were appropriate for their cognitive and motor abilities.

Exclusion criteria included a lifetime history or current diagnosis of serious orthopedic or neurological conditions (e.g., fractures of the upper extremities, congenital deformities, cerebral palsy, or musculoskeletal pain or dysfunction lasting more than two weeks), a history of surgical interventions, or recent musculoskeletal pain or dysfunction within one month prior to testing.

**Figure 2:**
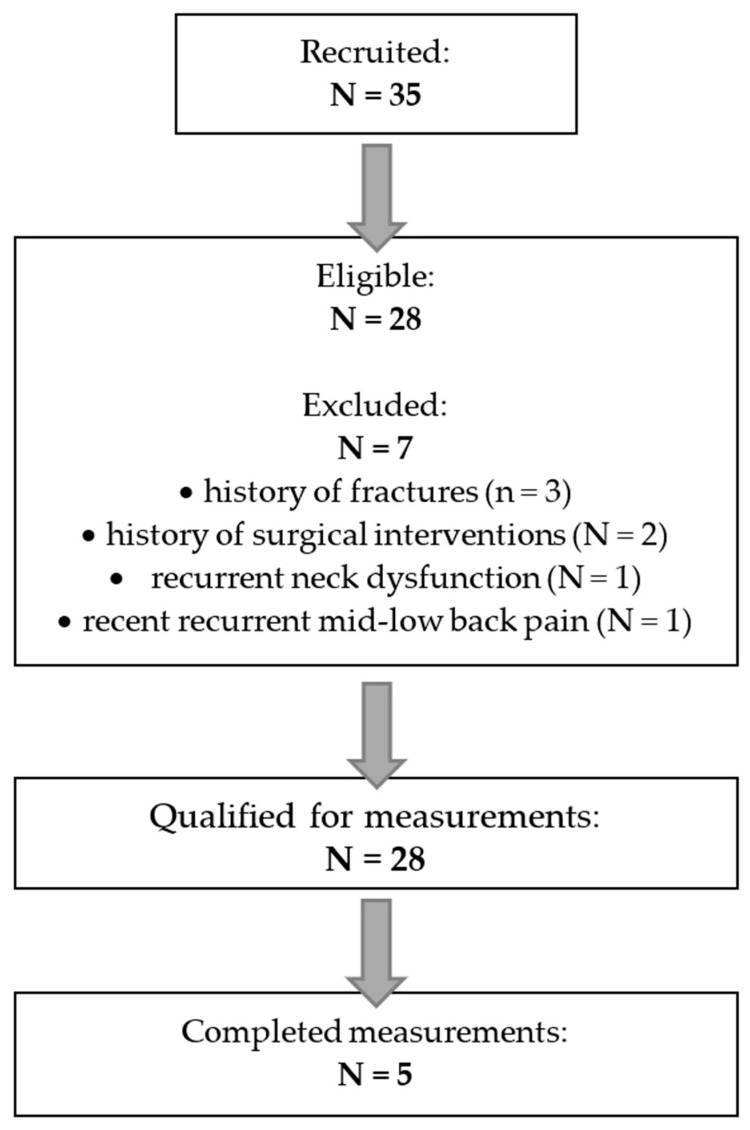
Flow of participants through the study.

During the one-month recruitment period, 35 children volunteered to participate. Seven volunteers were excluded due to a history of fractures (3), history of surgical interventions (2), recurrent neck dysfunction and pain (1), or recent recurrent mid- to lower-back pain that had not yet been diagnosed (1). A total of 28 children (12 girls, 42.9%; 16 boys, 57.1%) met the inclusion criteria and were enrolled in the study (Figure 2), exceeding the minimum required sample size (required n = 17; enrolled n = 28). The participants’ mean age was 12.86 years (range 10–15 years), mean body mass was 43.68 kg (range 31–58 kg), and mean height was 149.32 cm (range 138–165 cm). Twenty-three children (82.1%) were right-handed. All participants and their parents received detailed information about the study procedures, and written informed consent was obtained from the parents. Measurements were conducted in the Motion Analysis Laboratory at a local academic institution. No participants withdrew during the study.

### 2.2. Raters

Two experienced raters participated in the study. Rater A had seven years of clinical physiotherapy experience, including work with children and adolescents, and five years as technical staff in the Motion Analysis Laboratory, where they were responsible for conducting various measurements of human motor activity. Rater B had ten years of clinical physiotherapy experience and four years of laboratory work in the same setting. Both raters met the local professional requirements for physiotherapists working with children [46].

Both raters were actively involved in the development of the JPS and FS tests and possessed comparable, in-depth knowledge of the procedures and technical details. After the finalization of the test protocols, both raters underwent additional training to ensure precise, consistent, and efficient execution of all measurement procedures.

### 2.3. Measurements

Each participant completed four proprioceptive assessments: JPSrep, JPSdif, FSrep, and FSdif. All tests were conducted unilaterally, as bilateral JPS procedures may obscure the distinction between peripheral and central contributions to proprioceptive performance [47]. The non-dominant upper limb was assessed to minimize the influence of habitual motor use and learned movement patterns, providing a more sensitive measure of intrinsic sensory acuity that is less affected by skill-related adaptations. This approach also increased task difficulty, as measurements on the dominant limb would typically show slightly greater reliability [36,38,48].

The JPS and FS assessments were administered on two separate occasions. Within each session, JPSrep always preceded JPSdif, and FSrep preceded FSdif. The direction of shoulder movement (flexion or abduction) was randomized. Prior to formal testing, all participants completed a familiarization session in which they practiced each procedure using target values different from those employed during the main trials.

#### 2.3.1. Joint Position Sense Tests

For JPSrep, the passive acquisition–active reproduction paradigm was used to isolate the proprioceptive component of shoulder joint position sense [49,50,51,52]. This paradigm is particularly suitable for individuals with DCD, who often have difficulty planning and executing complex voluntary movements. By reducing the influence of motor planning deficits, it enables a more valid and reliable assessment of afferent-based JPS [40,45].

**Figure 3:**
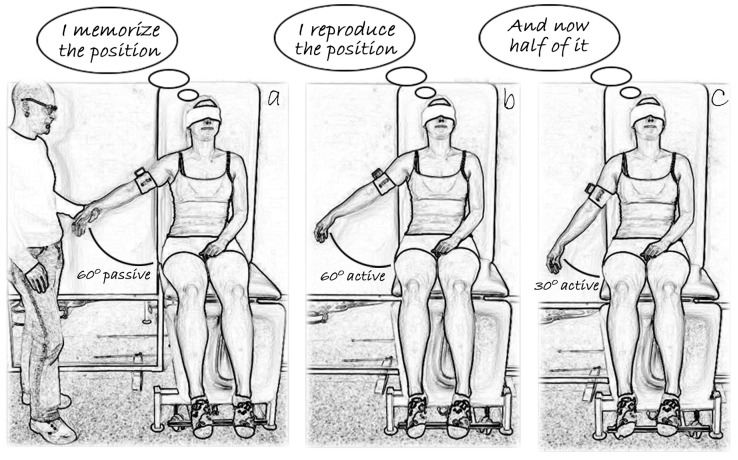
Assessment of joint position sense during shoulder abduction (for transparency): (**a**) memorization of the target angle (60 deg), during which the rater passively moves the participant’s shoulder to the target angle and holds it for 5 s; (**b**) join position reproduction test: the participant actively reproduces the target angle; and (**c**) joint position differentiation test: the participant reproduces half of the target angle. Joint position sense assessment during shoulder flexion was conducted in an analogous manner.

The task was performed using the K-Force Move v3 electronic goniometer (Kinvent Biomecanique, Montpellier, France), with position output recorded in degrees. The device was attached to the lateral aspect of the upper arm, midway between the acromion and lateral epicondyle. Participants sat in an adjustable chair with firm back support (Figure 3), with the tested limb hanging freely beside the trunk in a natural position (forearm supinated, thumb pointing forward, elbow flexed to approximately 10 deg). For shoulder flexion and abduction JPSrep, a target angle of 60 deg was selected to avoid end-range positions. Before each trial, the rater passively moved the participant’s shoulder to the target angle at a slow, constant speed of approximately 5 deg/s, holding the position for 5 s to allow memorization. The limb was then returned slowly to the starting position, from which the JPSrep trial commenced. Participants actively flexed or abducted the shoulder at a self-selected but slow speed and verbally indicated the perceived target position by saying “stop.” The reproduced angle was recorded for 3 s at a frequency of 30 Hz. Three trials were completed with 15 s intervals. Participants were blindfolded throughout to eliminate visual cues. Before each memorization phase and each reproduction trial, participants performed a short isometric co-contraction of the shoulder muscles to control for thixotropic effects in the musculotendinous tissues [53,54,55].

The JPSdif test followed the same procedure, except that participants were asked to reproduce “half of the target angle,” increasing cognitive load and emphasizing central processing of proprioceptive information.

#### 2.3.2. Force Sense Tests

For FSrep, the same passive acquisition–active reproduction paradigm was employed. Force sense was assessed using the K-Force Pull v3 electronic tensometer (Kinvent Biomecanique, Montpellier, France), with force output recorded in force kilograms. The device was secured at the midpoint of the upper arm using a Velcro strap and connected perpendicularly to the floor via a non-elastic rope. The setup was adjusted to allow isometric contraction of the shoulder flexors or abductors at 60°.

Prior to testing, participants performed three 5 s maximal voluntary isometric contractions (MVCs) for both flexion and abduction, each separated by at least one minute of rest. The mean of the three attempts served as the reference for calculating the 50% MVC target used in the FSrep test. The FS assessment began 10 min after completion of the MVCs [54,55]. Because force sense testing may elicit either perceived force or perceived effort, task instructions emphasized the perception of applied force rather than subjective exertion [56]. This ensured that performance primarily reflected afferent input from muscle spindles, Golgi tendon organs, and tactile cues from the strap–arm interface.

**Figure 4:**
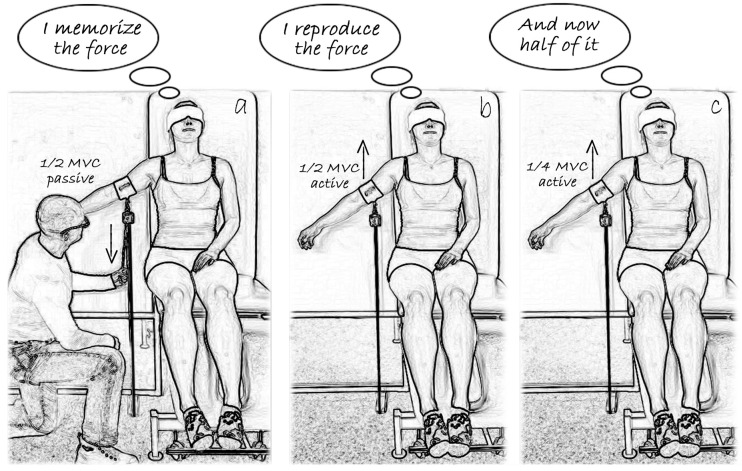
Assessment of force sense during shoulder abduction (for transparency): (**a**) memorization of the target force (50% of maximal voluntary contraction (MVC)), during which the rater applies the target force for 5 s; (**b**) force reproduction test: the participant actively reproduces the target force; and (**c**) force differentiation test: the participant generates a force equal to half of the target force. Force sense assessment during shoulder flexion was conducted in an analogous manner.

For FSrep (Figure 4), participants sat in the adjustable chair adopting the same resting upper-limb position used in the JPS tasks (forearm supinated, elbow flexed ~10 deg) and actively moved to 60 deg of shoulder flexion or abduction to achieve slight pre-tension of the strap–tensometer–rope setup. The rater then applied a slowly increasing downward force using an additional loop attached to the tensometer, aligned with the setup, until reaching 50% MVC. This load was held for 5 s to allow memorization. Participants were instructed to “hold the limb steady in this position” and “memorize the force required to maintain it.” After the load was released to zero, the reproduction trial commenced: participants generated an isometric contraction at a slow, self-selected rate and verbally indicated “stop” once they believed the target force had been reproduced. The produced force was recorded for 3 s at a frequency of 30 Hz. Three trials were performed with 15 s intervals. Participants were blindfolded throughout.

The FSdif test followed the same procedure, except that participants were instructed to reproduce “half of the target force,” emphasizing central processing.

### 2.4. Procedure

After completing the initial interview and confirming eligibility, parents and children selected two convenient measurement dates scheduled 7–10 days apart. All assessments were conducted in the stable laboratory conditions in the afternoon. Participants had to rest for at least three hours after school and to consume their last meal at least two hours before the given measurement session. They were asked to wear loose, non-restrictive clothing and completed a 10 min low-intensity warm-up on a stationary arm cycle. Participants were then briefed on test procedures and completed 4–5 familiarization trials of each task.

Raters were randomly assigned the identifiers A and B, and the testing order always followed the sequence A-B. During the first session, Rater A conducted series 1 of JPSrep and JPSdif tests. All equipment and skin markings were then removed, and participants rested for 15 min while sitting or walking within the laboratory without engaging in any excessive upper-limb activity. Rater A then prepared the participant again and performed series 2 of the tests. Following a further removal of equipment and markings and an additional 15 min rest period, Rater B conducted series 3 using the same procedure (Figure 1). During the second session, FSrep and FSdif were administered in an identical manner.

All measurements were carried out between May and June to maintain a consistent functional context, including comparable school demands and daily routines. Participants were instructed to continue their usual physical activity levels and avoid any abrupt changes throughout the measurement period.

To maintain blinding, a technical assistant was employed to record the measurement device outputs, which were concealed from the raters throughout the process.

### 2.5. Data Processing and Analysis

Data recorded during the test procedures were extracted and entered into a spreadsheet by the technical assistant who was not directly involved in the study.

Mean values from each 3 s recording window were calculated for all tests and used as input for subsequent analyses. For JPSrep and FSrep, absolute error was computed as │target value − reproduced value│, and for JPSdif and FSdif as │½ target value − produced differentiated value│.

In the first step of the statistical analysis, to compare group-level mean absolute errors across the three measurement series, a repeated-measures ANOVA was performed with measurement series as the repeated factor. Critical significance level was set at *p* < 0.05.

In the second step, intra-class correlation coefficients (ICCs) were calculated using model 2,*k* to permit generalization to a wider population of raters with similar characteristics. ICCs were computed separately for single repetitions of the JPS and FS tests, for the mean of two repetitions, and for the mean of three repetitions. Standard errors of measurement (SEM = SD × (1 − ICC)½) and smallest detectable differences (SDD = 1.96 × 2½ × SEM) were also derived. ICCs were interpreted as follows: 0.00–0.50 = poor reliability, 0.50–0.75 = moderate reliability, 0.75–0.90 = good reliability, and 0.90–1.00 = excellent reliability [57,58].

In the third step, Bland–Altman plots were generated to illustrate the individual as well as mean intra- and inter-rater bias, together with the corresponding limits of agreement.

The results are presented following the sequence of the statistical analyses. All analyses were performed using Statistica 13 (StatSoft Inc., Tulsa, OK, USA).

## 3. Results

Table 1 presents the descriptive statistics for the four tests used in the study, as assessed by two raters across consecutive measurement series. No significant differences were found between the series. Notably, absolute errors were considerably larger in the differentiation tests than in the reproduction tests—by a factor of approximately three to four.

The results of the JPS and FS tests demonstrated consistently high reliability across all conditions. This was evident both when measurements were repeated by the same rater and when they were performed by different raters (Table 2 and Table 3). For all tests and movement directions, the majority of ICCs fell within the excellent range, with only a small number of values dropping into the good range. The lowest ICC observed was 0.86, recorded for the inter-rater assessment of JPSrep in flexion based on a single measurement. Even in this instance, the associated SEM and SDD values remained acceptable (SEM = 1.20 deg, SDD = 3.32 deg). For intra-rater reliability, single measurements already produced ICCs above 0.90 for most conditions, indicating excellent reliability. Averaging two or three repeated measurements resulted in further increases, with ICCs typically reaching 0.97 or higher. This improvement was accompanied by progressive slight reductions in both SEM and SDD values, illustrating enhanced measurement precision with repeated trials. Inter-rater reliability was similarly robust. Even when only a single measurement was used, most ICCs exceeded 0.90. Averaged measurements further strengthened reliability, yielding ICCs frequently above 0.95 and producing lower SEM and SDD estimates.

**Figure 5:**
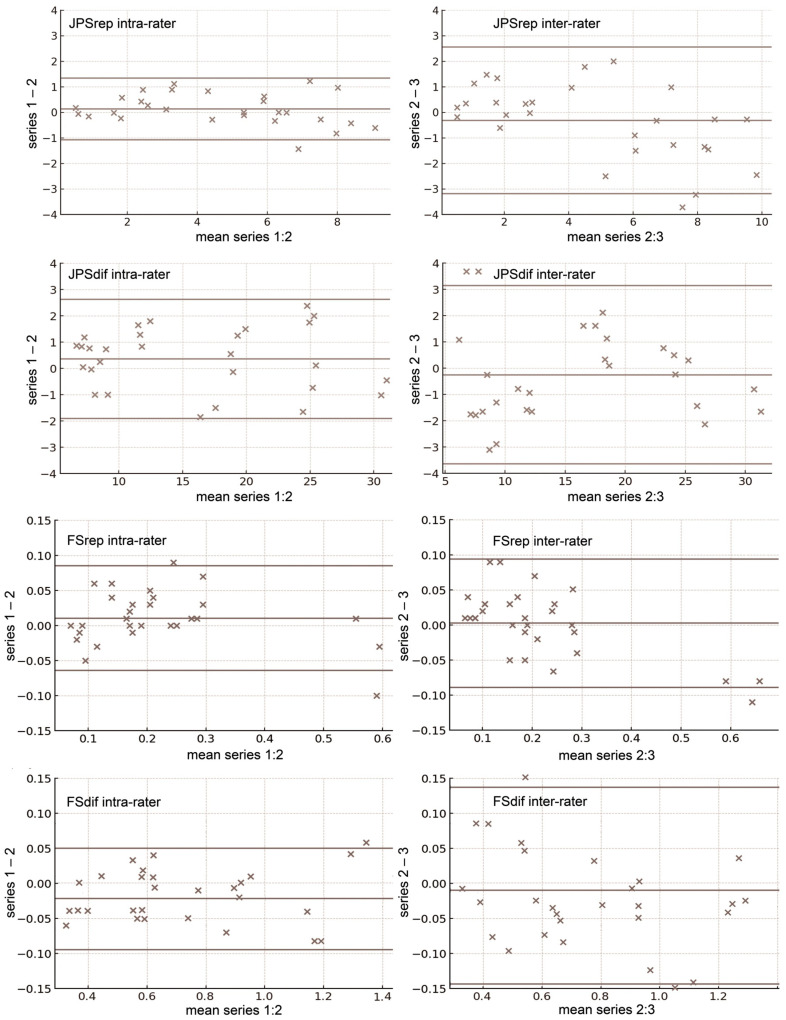
Based on three repeated measurements Bland–Altman plots for joint angle reproduction (JPSrep) and differentiation (JPSdif) tests, and force reproduction (FSrep) and differentiation (FSdif) tests. Plots are shown for intra-rater agreement (measurement series 1 vs. 2) and inter-rater agreement (series 2 vs. 3). The solid horizontal lines represent, from bottom to top, the lower limit of agreement, the mean difference between the compared measurement series, and the upper limit of agreement. The plots for shoulder flexion are considered representative of those obtained for shoulder abduction.

Analysis of the Bland–Altman plots indicates that the differences between the two consecutive measurement series, whether obtained by the same rater (series 1 vs. 2) or by two different raters (series 2 vs. 3), showed a fair degree of concentration around the mean and generally fell within the limits of agreement, with only occasional outliers, and with mean differences close to zero. This pattern was consistent across all tests and both movement directions (Figure 5).

## 4. Discussion

The present study demonstrated that the modified JPS and FS protocols achieved consistently high reliability across all measurement conditions in children with symptoms of DCD. Both intra- and inter-rater reliability indices reached levels that support their use in research contexts aiming to incorporate clinically oriented proprioceptive assessments. Such reliability was achieved despite the well-documented challenges faced by children with DCD, including reduced attentional capacity, working-memory limitations, and altered sensory processing [25,30,45]—factors that frequently complicate the administration of proprioceptive tasks. Moreover, the high reliability of our measurements indicates that, when appropriately adapted, JPS and FS assessments can be used confidently in studies where advanced technological solutions (e.g., motion-tracking systems) may be impractical, unavailable, or overly restrictive.

Achieving this level of reliability required several targeted modifications to the traditional adult-oriented protocols. These adjustments were informed by pilot testing, which revealed that standard procedures [54,55] impose excessive cognitive and attentional demands on children with DCD, potentially compromising the stability of their performance. To address these challenges, the assessments were conducted across two separate days to reduce fatigue and promote steady performance. The familiarization phase was extended to up to five repetitions to support clearer understanding and more robust encoding of target positions and forces. Target exposure duration was increased to 5 s to facilitate proprioceptive memorization, while longer rest periods (3 min) were implemented between tests in different movement directions. Inter-trial intervals were shortened to 15 s to help maintain attention throughout the tasks. Child-appropriate language was used to improve clarity and comprehension, and stabilization straps were omitted due to poor tolerance and potential effects on comfort and engagement. Collectively, these adaptations enhanced the feasibility, ecological validity, and reliability of the testing procedures while preserving the core structure of the original protocols.

Unfortunately, there is limited literature available to directly compare our findings with. The reliability levels observed in the present study correspond closely with, and in several instances exceed, those reported in adult samples using standardized JPS and FS protocols. For example, Goble [36] and Goble & Brown [48] reported intra-rater ICCs for upper-limb JPS reproduction in healthy adults ranging from 0.88 to 0.95, with SEMs between 0.5 and 1.5 deg, while Niespodziński et al. [54,55] found ICCs of 0.91–0.98 and SEMs of 0.3–1.2 deg for both JPS and FS tasks. Dover et al. [59] reported inter-rater ICCs of 0.85–0.96 for shoulder JPS and force reproduction measures. In comparison, the present study achieved intra-rater ICCs of 0.92–0.99 for JPSrep and 0.92–0.99 for FSrep in children with DCD, with SEMs generally below 1.1 deg for JPS and 0.08 force kilograms for FS, and inter-rater ICCs ranging from 0.86 to 0.99. These values indicate that the adapted protocols yielded reliability levels comparable to or exceeding those obtained in adults under controlled conditions.

This finding is particularly noteworthy because children with DCD commonly exhibit cognitive and sensorimotor challenges [25,30,45] that can impair task performance, along with possible molecular and structural alterations of the central nervous system [23,24]. Previous studies in children have reported lower or more variable reliability in similar assessments: for instance, Holst-Wolf et al. [31] documented ICCs of 0.75–0.88 for forearm JPS in typically developing children, and Law et al. [26] reported greater variability in FS measures in children with DCD. Against this backdrop, the present findings provide novel evidence that reliable proprioceptive and force sense measurements are achievable in this population when protocols are thoughtfully adapted to meet their functional and cognitive needs. The procedural modifications implemented—including extended familiarization, longer target exposure, structured rest intervals, child-friendly instructions, and the removal of poorly tolerated stabilization straps—appeared to support stable performance across sessions and raters, confirming their practical utility for future research in children with DCD.

In our results, we chose to present the ICC model 2,*k* to enable generalization to broader populations of similar raters. By using this model the reported reliability reflects not only the reliability of a test performed by a specific rater but also the expected reliability across other raters with similar training and experience, providing a more realistic estimate for research and clinical settings. Naturally, the ICC model 3,*k*, which reflects the reliability of measurements performed by a specific rater(s), would yield higher values, as it excludes variability attributable to differences between raters and is therefore less generalizable [57,58].

To enhance the practical applicability of the JPS and FS tests, the present results pro-vide quantitative guidance on both testing protocols and clinical interpretation. Within a scientific context, we recommend obtaining three repeated measurements per test, particularly when different raters perform the test and retest, as this reduces variability attributable to rater effects and yields narrower confidence intervals, lower SEMs, and smaller SDDs For example, in the JPSrep flexion task, the inter-rater SDD decreases from 3.32 deg with a single measurement to 2.10 deg when three repetitions are averaged (Table 2). When a single, consistent rater conducts all assessments, two repetitions are generally sufficient, although adding a third repetition requires minimal extra effort and further improves measurement stability. Importantly, SEM and SDD values can directly inform clinical decision-making by indicating the magnitude of change required to exceed measurement error. For instance, in the example above, changes smaller than ~2 deg may reflect measurement noise rather than true differences. These values provide clinicians with practical thresholds for interpreting whether a change between assessments represents a meaningful change in JPS rather than random variability.

From an implementation perspective, these protocols can be readily applied outside laboratory settings, as they require minimal staff, equipment, and time. In routine clinical practice, two repetitions may be sufficient when testing conditions are stable and the same examiner performs repeated assessments, while three repetitions are advisable when different clinicians are involved or when higher diagnostic confidence is required. Where the child’s condition and cooperation allow, including additional repetitions (up to four or five) may further reduce the influence of occasional outliers and improve measurement stability, which is particularly relevant when individual-level decisions are made. Conversely, reducing the number of repetitions below two is not recommended, as single measurements increase susceptibility to random error and may lead to misinterpretation of small changes.

While joint position and force reproduction tasks are well established in both adult and pediatric populations [34,54,55,56], the differentiation tasks employed in the present study—reproducing half of a memorized joint angle (JPSdif) or target force (FSdif)—remain relatively novel. Unlike traditional JPS and FS assessments, which primarily reflect peripheral proprioceptive input from muscle spindles, Golgi tendon organs, and joint mechanoreceptors [47,56], differentiation tasks introduce an additional cognitive component that requires participants to scale and transform a previously memorized stimulus. Conceptually, this aligns with psychophysical threshold tests and dual joint position paradigms [32,36], which emphasize central integration of proprioceptive signals rather than simple peripheral encoding. Successful performance on JPSdif and FSdif likely depends on higher-order cognitive mechanisms, such as maintaining the proprioceptive stimulus in working memory, scaling the remembered amplitude, and making proportional reproduction decisions. For instance, proprioceptive memory retention is reduced under competing verbal and spatial loads, indicating reliance on working memory resources when reproducing joint positions [60]. Moreover, somatosensory working memory capacity predicts performance in motor tasks requiring recall of learned limb movements, further linking memory systems with proprioceptive-motor integration [61]. Neuroimaging and lesion studies also show that position sense engages distributed cortical and subcortical networks, including posterior parietal and sensorimotor regions, beyond simple peripheral encoding [62]. Finally, attentional modulation alters proprioceptive processing in primary sensorimotor cortex, suggesting that cognitive state and network involvement influence proprioceptive judgments [63]. In practice, some participants found the JPSdif task challenging: when asked to reproduce half of the target angle (30 deg), occasional absolute errors (>30 deg; Table 1) approached or even exceeded the full target angle (60 deg). This suggests that task comprehension, central scaling of memorized proprioceptive input, or working memory limitations may contribute to performance variability.

Despite mentioned large absolute errors, reliability metrics remained high, indicating consistent measurement precision. However, although JPSdif and FSdif demonstrate high reliability, their theoretical and clinical validity is not yet fully established. Studies comparing different proprioceptive assessments show that common position-matching paradigms [32] and psychophysical threshold measures capture distinct physiological processes: threshold methods rely on passive motion and predominantly reflect sensory afferent processing, whereas active matching tasks engage additional sensorimotor integration, including working memory, motor planning, and interhemispheric communication, which may not directly index ‘pure’ proprioceptive acuity [32]. These findings suggest that active differentiation tasks like JPSdif/FSdif could be influenced by multiple overlapping mechanisms, complicating interpretation unless their specific contributions are better characterized. The exact modality or cognitive element assessed by these differentiation tasks is yet not fully understood, highlighting the need for further validation. These findings underscore that while differentiation tasks may offer enhanced sensitivity for detecting subtle proprioceptive or central processing deficits, careful methodological refinement and theoretical grounding are required before they can be confidently applied in experimental or clinical contexts.

A side observation in the present study was the relatively high number of unsuccessful trials, particularly in the FS tasks. Participants were asked to reproduce 50% (FSrep) and 25% (FSdif) of their maximal voluntary contraction, yet up to six out of nine trials (3 series × 3 trials) were unsuccessful. Across the cohort, the average number of failed FS trials was 3.2 ± 1.3 per nine attempts (range 0–6), corresponding to 35.6% ± 14.4% (range 0–67.7%). By contrast, unsuccessful trials were less frequent in the JPS tasks, averaging 1.7 ± 0.6 per nine attempts (18.9% ± 6.7%, range 0–3.3%). In the FS tasks, the most common reason for failure was continuing to increase force after the ‘stop’ command. Of course, some of the unsuccessful trials were due not to the child’s performance but to technical issues, such as movement of the equipment connections or occasional data transmission failures. These occurrences, however, were distributed fairly evenly between the JPS and FS tasks. Although these observations were not reported in the Section 3, as they are not directly related to the study objectives, we felt it important to mention them here to provide context for interpreting the reliability outcomes.

The high rate of unsuccessful FS trials likely reflects the specific challenges children with DCD face in force modulation, motor planning, and sustained attention, rather than excessive task demands, which were intentionally moderate. Repeated attempts may have improved familiarization and contributed to more stable performance over time, but they could also have introduced subtle practice effects, potentially inflating reliability estimates. These findings underscore the importance of providing sufficient practice, clear instructions, and flexible protocol adaptations when assessing children with DCD, especially in tasks requiring precise force control. Reporting the frequency and distribution of unsuccessful trials helps contextualize the reliability results and informs expectations for both research and clinical assessment.

In summary, the present study demonstrates that JPSrep and FSrep tests can be administered reliably in children with DCD and are now ready to be implemented in other studies including our planned experimental study investigating the effects of an intensified sensory stimulation intervention. In contrast, the JPSdif and FSdif tests require further investigation regarding their validity, before they can be confidently applied in experimental or clinical contexts. These findings provide a solid methodological foundation for future research evaluating proprioceptive function in pediatric populations with motor difficulties.

### Limitations

Several limitations of the present study should be acknowledged. First, the sample size was relatively small and drawn from a narrow population of adolescents with DCD, although it exceeded the minimum recommended for reliability analysis by nearly 50%. This focus was intentional, as the primary aim was to establish measurement reliability specifically within the DCD population, which will serve as the basis for subsequent experimental studies. Nevertheless, caution is warranted when generalizing these findings to broader populations or different age groups. Second, movement directions were not fully randomized; tests for one direction (flexion or abduction) were consistently performed before the other. This fixed order was adopted to minimize cognitive load and motor planning demands in children with DCD, for whom frequent task switching and unpredictable sequencing can negatively affect task execution and compliance. While randomization or counterbalancing could theoretically reduce potential order effects, these approaches may introduce additional variability related to attentional and organizational difficulties rather than true measurement error in this population. Future studies may nevertheless explore partial randomization or counterbalancing to further evaluate the robustness of this methodological choice. Third, a relatively high number of unsuccessful trials occurred, particularly in the FS tasks, which may have increased familiarization effects and slightly inflated reliability estimates.

Despite these limitations, the modified procedures offer practical advantages. They require limited time, personnel, and equipment and can be conducted by a single examiner (the assistant was used only for blinding). Equipment costs are modest, relying on two small digital devices that are commonly available in clinical settings, in contrast to more complex motion analysis systems used in other studies [34,52,53,57]. Future research should address the above limitations by expanding sample size, refining task protocols to reduce unsuccessful trials (e.g., extended practice, alternative feedback, or adjusted force levels), and further examining the impact of task order.

## 5. Conclusions

This study provides the first systematic evidence that JPS and FS assessments can be administered with high intra- and inter-rater reliability in children with DCD. Consistently excellent ICCs and low SEM and SDD values confirm strong measurement precision, directly addressing the primary objective of establishing reliability. The recommended use of at least three repetitions, together with protocol adaptations (extended familiarization, longer target exposure, child-appropriate instructions, and adjusted rest intervals), ensured feasibility and reliability while accommodating the attentional and motor planning challenges characteristic of DCD.

Importantly, the demonstrated reliability supports the use of JPS and FS measures as outcome tools for evaluating targeted therapeutic interventions aimed at improving proprioceptive function and motor control, fulfilling the second study objective.

While the differentiation tasks (JPSdif and FSdif) showed high reliability, their validity requires further investigation. Overall, this study establishes a reliable and developmentally appropriate framework for proprioceptive and force assessment in children with DCD, with future research warranted to confirm responsiveness to intervention and to further validate differentiation tasks.

## Figures and Tables

**Table 1:** Mean absolute errors (±standard deviation (minimum-maximum)) calculated for the three repeated measurements of the joint angle reproduction (JPSrep) and joint angle differentiation (JPSdif) tests (in degrees), as well as in the force reproduction (FSrep) and force differentiation (FSdif) tests (in force kilograms). The *p* values and effect sizes (η) for inter-series differences (Anova for repeated measurements) are included.

	Flexion		Abduction	
Rater/Series	JPSrep	JPSdif	JPSrep	JPSdif
1/1 (a)	4.71 ± 2.53 (0.57–8.78)	16.29 ± 8.09 (7.10–31.98)	4.15 ± 3.51 (0.89–13.72)	16.62 ± 10.08 (6.16–40.83)
1/2 (b)	4.57 ± 2.69 (0.44–9.39)	15.91 ± 8.22 (6.23–32.42)	3.95 ± 3.40 (0.91–14.72)	16.83 ± 10.34 (5.06–42.89)
2/3 (c)	4.89 ± 3.48 (0.44–11.06)	16.15 ± 8.28 (4.93–34.06)	3.96 ± 3.99 (0.39–15.33)	16.96 ± 9.39 (4.78–39.39)
*p* (η) Anova	0.42 (0.03)	0.48 (0.03)	0.55 (0.02)	0.47 (0.03)
	**FSrep**	**FSdif**	**FSrep**	**FSdif**
1/1 (a)	0.23 ± 0.14 (0.07–0.58)	0.72 ± 0.30 (0.23–1.36)	0.21 ± 0.13 (0.04–0.57)	0.78 ± 0.28 (0.31–1.31)
1/2 (b)	0.22 ± 0.15 (0.07–0.64)	0.74 ± 0.30 (0.32–1.30)	0.21 ± 0.13 (0.03–0.54)	0.77 ± 0.26 (0.41–1.25)
2/3 (c)	0.21 ± 0.18 (0.05–0.72)	0.76 ± 0.34 (0.33–1.54)	0.21 ± 0.13 (0.03–0.57)	0.78 ± 0.26 (0.37–1.18)
*p* (η) Anova	0.32(0.04)	0.10 (0.08)	0.59 (0.02)	0.92 (0.01)

**Table 2:** Intra- and inter-rater reliability estimates for the absolute errors in the joint angle reproduction test (JPSrep) and joint angle differentiation test (JPSdif). Presented are: intraclass correlation coefficient (ICC (±95% confidence interval); models: 2,1 for single measurements 2,2 for two repeated measurements, and 2,3 for three repeated measurements), standard error of measurement (SEM (in degrees)) and smallest detectable difference (SDD (in degrees)).

	Reliability	N Measurements	Flexion			Abduction		
			ICC (±95%CI)	SEM	SDD	ICC (±95%CI)	SEM	SDD
JPSrep	intra-rater	1	0.92 (0.83–0.96)	0.76	2.10	0.96 (0.92–1.00)	0.72	1.99
		2 (mean)	0.98 (0.93–1.00)	0.36	1.01	0.97 (0.94–0.99)	0.61	1.68
		3 (mean)	0.99 (0.96–1.00)	0.26	0.72	0.97 (0.94–0.99)	0.60	1.66
	inter-rater	1	0.86 (0.76–0.93)	1.20	3.32	0.95 (0.91–0.99)	0.86	2.37
		2 (mean)	0.93 (0.88–0.97)	0.83	2.29	0.98 (0.96–1.00)	0.53	1.48
		3 (mean)	0.94 (0.90–0.97)	0.76	2.10	0.97 (0.95–1.00)	0.64	1.77
JPSdif	intra-rater	1	0.99 (0.96–1.00)	1.08	2.99	0.92 (0.87–0.96)	3.00	8.31
		2 (mean)	0.99 (0.97–1.00)	0.84	2.32	0.99 (0.96–1.00)	1.05	2.90
		3 (mean)	0.99 (0.97–0.99)	0.81	2.24	0.99 (0.96–0.99)	1.02	2.82
	inter-rater	1	0.98 (0.95–0.99)	1.53	4.23	0.94 (0.90–0.99)	2.51	6.96
		2 (mean)	0.99 (0.96–0.99)	0.85	2.35	0.98 (0.95–1.00)	1.41	3.92
		3 (mean)	0.99 (0.97–1.00)	0.82	2.27	0.99 (0.96–0.99)	0.98	2.71

**Table 3:** Intra- and inter-rater reliability estimates for the absolute errors in the force reproduction test (FSrep) and force differentiation test (FSdif). Presented are: intraclass correlation coefficient (ICC (±95% confidence interval); models: 2,1 for single measurements 2,2 for two repeated measurements, and 2,3 for three repeated measurements), standard error of measurement (SEM (in force kilograms)) and smallest detectable difference (SDD (in force kilograms)).

	Reliability	N Measurements	Flexion			Abduction		
			ICC (±95%CI)	SEM	SDD	ICC (±95%CI)	SEM	SDD
FSrep	intra-rater	1	0.92 (0.82–0.98)	0.04	0.12	0.94 (0.88–0.98)	0.03	0.09
		2 (mean)	0.97 (0.91–0.99)	0.03	0.07	0.99 (0.96–1.00)	0.03	0.08
		3 (mean)	0.98 (0.95–0.99)	0.02	0.06	0.99 (0.96–0.99)	0.01	0.04
	inter-rater	1	0.96 (0.92–0.99)	0.03	0.09	0.93 (0.86–0.97)	0.04	0.10
		2 (mean)	0.98 (0.96–1.00)	0.02	0.07	0.98 (0.96–1.00)	0.02	0.05
		3 (mean)	0.98 (0.96–0.99)	0.02	0.07	0.99 (0.96–0.99)	0.01	0.04
FSdif	intra-rater	1	0.98 (0.94–1.00)	0.04	0.12	0.92 (0.86–0.97)	0.08	0.21
		2 (mean)	0.99 (0.96–1.00)	0.03	0.09	0.98 (0.94–1.00)	0.04	0.11
		3 (mean)	0.99 (0.97–1.00)	0.03	0.08	0.98 (0.96–0.99)	0.04	0.11
	inter-rater	1	0.91 (0.85–0.98)	0.10	0.27	0.93 (0.87–0.97)	0.07	0.18
		2 (mean)	0.96 (0.93–1.00)	0.07	0.18	0.97 (0.94–0.99)	0.04	0.12
		3 (mean)	0.98 (0.96–0.99)	0.04	0.12	0.98 (0.96–0.99)	0.04	0.10

## Data Availability

The original contributions presented in this study are included in the article. Further inquiries can be directed to the corresponding author.

## Abbreviations

The following abbreviations are used in this manuscript:

DCD	Developmental Coordination Disorder
FS	Force sense
FSdif	Force differentiation test
FSrep	Force reproduction test
JPS	Joint position sense
JPSdif	Joint angle differentiation test
JPSrep	Joint angle reproduction test
